# Role of GirK Channels in Long-Term Potentiation of Synaptic Inhibition in an In Vivo Mouse Model of Early Amyloid-*β* Pathology

**DOI:** 10.3390/ijms20051168

**Published:** 2019-03-07

**Authors:** Irene Sánchez-Rodríguez, Agnès Gruart, José María Delgado-García, Lydia Jiménez-Díaz, Juan D. Navarro-López

**Affiliations:** 1 Neurophysiology & Behavior Laboratory, Centro Regional de Investigaciones Biomédicas, School of Medicine of Ciudad Real, University of Castilla-La Mancha, 13071 Ciudad Real, Spain; irene.sanchez@uclm.es; 2 Division of Neurosciences, University Pablo de Olavide, 41013 Seville, Spain; agrumas@upo.es (A.G.); jmdelgar@uclm.es (J.M.D.-G.)

**Keywords:** A*β*_1–42_, synaptic plasticity, inhibitory LTP, GirK channels, neuronal excitability, hippocampus, fIPSP, in vivo, freely moving mice

## Abstract

Imbalances of excitatory/inhibitory synaptic transmission occur early in the pathogenesis of Alzheimer’s disease (AD), leading to hippocampal hyperexcitability and causing synaptic, network, and cognitive dysfunctions. G-protein-gated potassium (GirK) channels play a key role in the control of neuronal excitability, contributing to inhibitory signaling. Here, we evaluate the relationship between GirK channel activity and inhibitory hippocampal functionality in vivo. In a non-transgenic mouse model of AD, field postsynaptic potentials (fPSPs) from the CA3–CA1 synapse in the dorsal hippocampus were recorded in freely moving mice. Intracerebroventricular (ICV) injections of amyloid-*β* (A*β*) or GirK channel modulators impaired ionotropic (GABA_A_-mediated fPSPs) and metabotropic (GirK-mediated fPSPs) inhibitory signaling and disrupted the potentiation of synaptic inhibition. However, the activation of GirK channels prevented A*β*-induced changes in GABA_A_ components. Our data shows, for the first time, the presence of long-term potentiation (LTP) for both the GABA_A_ and GirK-mediated inhibitory postsynaptic responses in vivo. In addition, our results support the importance of an accurate level of GirK-dependent signaling for dorsal hippocampal performance in early amyloid pathology models by controlling the excess of excitation that disrupts synaptic plasticity processes.

## 1. Introduction

Alzheimer’s disease (AD) is a progressive neurodegenerative disorder that constitutes the leading cause of dementia. The main pathological hallmarks of AD are A*β*-containing extracellular senile plaques, and intracellular neurofibrillary tangles consisting of hyperphosphorylated tau protein. Hippocampal dysfunction is one of the first events observed during AD, and the hippocampus is subject to progressive atrophy throughout the course of the disease [1,2]. The correct functionality of hippocampal neuronal circuits depends on the balance between excitatory and inhibitory synaptic transmission, which is affected by the action of A*β* [3,4]. In early stages of AD, hippocampal hyperexcitability is observed [5,6], as well as other dysfunctions, such as the alteration of hippocampal oscillatory activity [7,8,9] and the inhibition of LTP, as well as the loss of synaptic plasticity in the intrahippocampal and cortical hippocampal tracts, due to soluble forms of A*β* [10,11,12]. All these factors ultimately lead to the deterioration of cognitive functions.

The G-protein-gated inwardly rectifying potassium (Kir3/GirK) channels are tetramers, conformed by four essential subunits (GIRK1, GIRK2, GIRK3 and GIRK4) [13,14]. In the central nervous system, GIRK1-3 are widely expressed, whereas the expression of GIRK4 is limited to only some neuronal populations, and does not contribute to GirK brain currents [15,16,17]. GIRK1/GIRK2 heteromultimers are the prototypic GirK channel in hippocampal neurons [16,18,19,20]. GirK channels are involved in regulating the firing of neurons, the resting potassium conductance and the membrane potential, thereby contributing to the suppression of neuronal hyperexcitability [21,22,23]. GirK channels also mediate the inhibitory effects of several neurotransmitters and neuromodulators, including GABA, serotonin, adenosine, dopamine, opioids, and somatostatin [24], thanks to the interaction with G-protein-coupled receptors (GPCR), so their activity is critical for synaptic plasticity in the dorsal hippocampus [25].

It has been suggested that restoration—through an increase in inhibitory signaling—of the excitatory and inhibitory balance impaired by A*β* could prevent neuronal dysfunction and the cognitive deficits associated with the early stages of AD [26,27,28]. We previously reported that an increase in GirK channel activity restores hippocampal activity at the synaptic, network, and cognitive levels in an in vivo mouse model of AD [29]. However, although the role of GirK-dependent signaling on the long-term potentiation (LTP) of synaptic inhibition has been linked in vitro to plasticity and network activity [30], no attention had been paid to its functional contribution to synaptic inhibition in vivo. Here, we have investigated the inhibitory components (inhibitory field postsynaptic potentials, fIPSPs) of the CA3–CA1 synapse. For that purpose, and for the first time, synaptic plasticity of fIPSPs has been studied in the dorsal hippocampus of freely moving mice, and the effect of A*β* and GirK modulation on such synaptic responses was analyzed with respect to inhibitory activity. Our data suggest that GirK channels are needed for adjusting GABA_A_ signaling to the excitatory activity, as well as for the LTP of synaptic inhibition. In addition, GirK activation improves hippocampal inhibitory activity disrupted by A*β*. Our results support the importance of an accurate level of GirK-dependent signaling for the LTP process in the dorsal hippocampal area, by controlling the excess of excitation that disrupts synaptic plasticity in early amyloid pathology models.

## 2. Results

**Figure 1:**
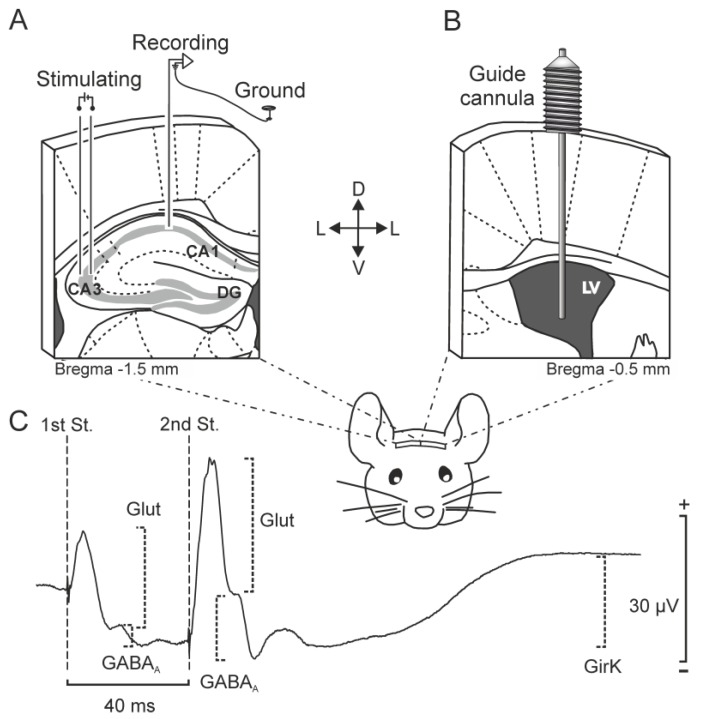
Experimental design. (**A**) The picture illustrates how mice were prepared for chronic recording of fEPSPs evoked at the hippocampal CA3–CA1 synapse, by surgical implantation of bipolar stimulating electrodes on the right Schaffer collaterals, and bipolar recording electrodes at ipsilateral CA1 region. A bare silver wire was fixed to the skull to act as ground. (**B**) The picture illustrates how mice were implanted with a stainless-steel guide cannula for drug administration. The cannula was implanted on the left ventricle, contralaterally to both electrodes, in order to preserve the functionality of CA3–CA1 synapse. (**C**) Representation of the fPSPs evoked in the CA1 hippocampal region after paired-pulse stimulation (interval of 40 ms) of the Schaffer collaterals. The recording was obtained from a representative animal and illustrates the averaged (*n* = 50) profile of the postsynaptic response. Three different components were identified for amplitude analysis: (1) a glutamatergic fEPSP, with a latency of appearance of 2.25–4 ms after stimulation, (2) a GABAergic fIPSP dependent on GABA_A_ receptors, with a latency of 12–15 ms, and (3) an fIPSP dependent on metabotropic receptors and GirK channels, with a latency of 26–36 ms. For each component or postsynaptic potential, the maximum amplitude (peak-to-peak value) was measured for the analysis. LV, Lateral Ventricle; DG, Dentate gyrus; St., stimulus; D, dorsal; M, medial; L, lateral; Glut, glutamate.

As detailed in the Methods section, animals were prepared for the chronic recording of fPSPs at CA3–CA1 synapses and for ICV injections (Figure 1A,B). Both recordings and injections were performed in freely moving animals. The relationship between GirK channel activity and inhibitory hippocampal functionality was analyzed in vivo in this model of early AD-like physiopathology. For this purpose, both inhibitory activity and inhibitory synaptic plasticity were studied in the CA3–CA1 synapse after ICV injections of selective GirK modulators or A*β*_1–42_, as previously described [29] to generate a mouse model of early amyloid pathology.

### 2.1. GABA_A_-Dependent Signaling is Disrupted by Aβ or GirK Blockage

In order to study the functional capabilities of the CA3–CA1 synapse in freely moving mice, an fPSP was evoked in the CA1 pyramidal cells by the electrical stimulation of Schaffer collaterals. Three clearly defined components could be identified in the fPSP (Figure 1C). The start latency measured for each one was as follows: glutamatergic fEPSP, 2–5 ms; GABA_A_-dependent fIPSP, 12–15 ms; and GirK-mediated fIPSP, 26–32 ms. This classification was done based on previous in vitro [31] and in vivo [32,33] data.

**Figure 2:**
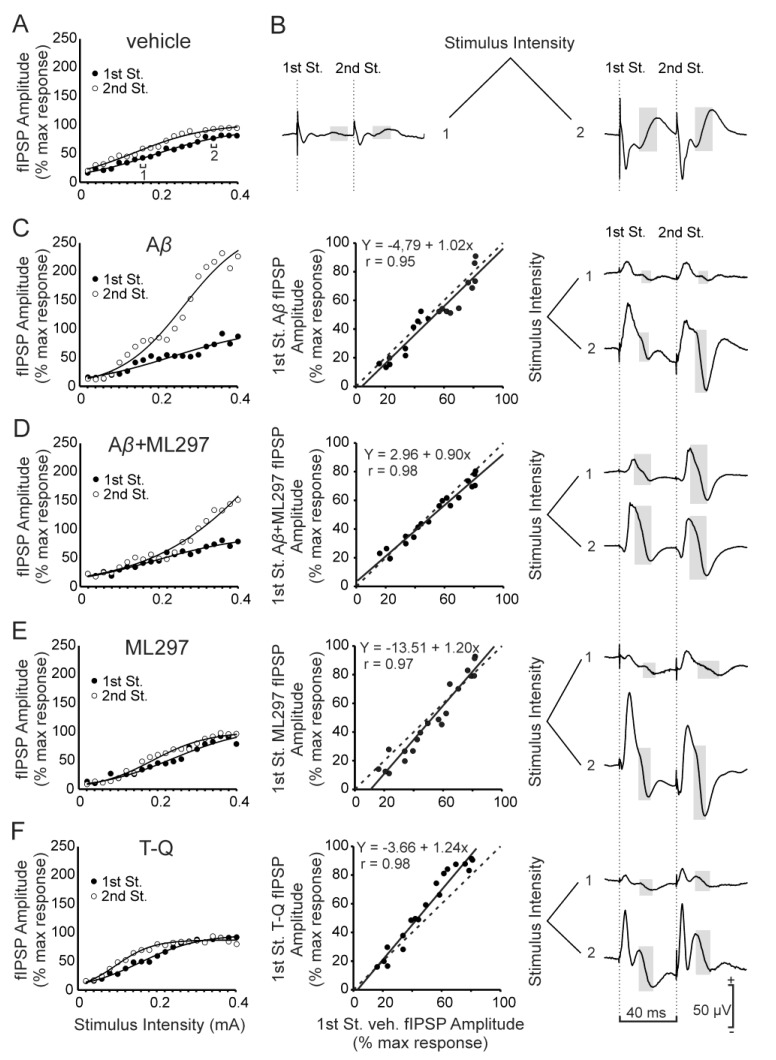
I/O curves for the GABA_A_-receptor-dependent inhibitory potential. (**A**) and left panel of (**C**–**F**), Relationship between the intensity (mA) of pairs of stimuli (40 ms interval between stimuli) applied to the Schaffer collaterals and the amplitude of the ionotropic GABAergic fIPSPs evoked in the CA1 region, corresponding to the first (black circles) and second (white circles) pulse. For each intensity of stimulation, circles represent the average of the response for all the animals of each experimental group. To facilitate the interpretation of the data, error bars have been omitted and the best sigmoid fit to the data for each group of animals has been illustrated (r ≥ 0.98 in all cases except for the 1st pulse of the A*β* group, r = 0.955; *p* < 0.001). Note the increase in the fIPSP amplitude caused by the 2nd stimulus in animals injected with A*β* (**C**) and how the drug ML297 was able to restore this effect to control (vehicle) levels. Animals injected with T-Q (**F**) did not present an increased amplitude in the 2nd pulse. Center panel of (**C**–**F**), Scatter plots and linear fits (continuous black lines) illustrating the amplitude values of fIPSP evoked by the first pulse in each experimental group vs. vehicle (control) group (x-axis, vehicle; y-axis, experimental group). Dashed lines represent the linear fit for the control conditions (vehicle vs. vehicle) and are equal in (**C**–**F**). The values of the slopes for these linear fits were compared with the control to study changes in the activity or tone of this inhibitory synaptic potential. (**B**) and right panel of (**C**–**F**) averaged recordings (*n* = 5) representative of the fIPSPs evoked in the CA1 area after stimulation of the ipsilateral Schaffer collaterals with pairs of pulses (interval between stimuli of 40 ms) at two different intensities (1, 0.16 mA; 2, 0.34 mA; the intensities 1 and 2 are indicated in (**A**)). The highlighted area marks the ionotropic GABAergic fIPSP subject of the analysis illustrated in this figure. St., stimulus.

Thereafter, we began our study of inhibitory synaptic transmission in the dorsal hippocampus by recording changes in amplitude of GABA_A_-dependent fIPSPs evoked in the pyramidal CA1 area by paired-pulse (40 ms interval) stimulation in vehicle-injected and drug-injected mice (Figure 2A,C–F left panel). To perform the analysis, we represented scatter plots and linear fits of the amplitude values for fIPSP evoked by the first pulse in each experimental group vs. the amplitude value for fIPSP evoked by the first pulse for the vehicle (control) group (Figure 2C–F, center panel; x-axis, vehicle; y-axis, experimental group). In control mice (vehicle, *n* = 14), the amplitude of the fIPSPs evoked in CA1 by the first and second pulses increased steadily with stimulus intensity (1st stimulus, F_(19, 247)_ = 16.67, *p* < 0.001, 2nd stimulus F_(19, 247)_ = 9.62, *p* < 0.001). The second stimulus presented a slightly larger amplitude than the first one (non-significant differences, F_(1, 26)_ = 3.07, *p* > 0.05) (see Figure 2A). In contrast, A*β*-injected animals showed a difference in the fIPSP between the 1st and 2nd stimulus, the latter displaying a significantly larger amplitude (F_(1, 12)_ = 10.89, *p* = 0.006, Figure 2C, *n* = 7). These results suggest that A*β* injections induced an increase in the inhibitory activity mediated by GABA_A_ receptors in CA1. However, the linear fit in the scatter plot (Figure 2C, center panel) had a slope close to 1 (b = 1.02, *p* < 0.001), so the evolution of the response to the 1st stimulus is not altered by the effect of A*β* in comparison to controls (vehicle-injected mice). Overall, the results indicate that these animals show an increase in the inhibitory tone.

Activation of GirK channels with the selective GIRK1-subunit-containing opener ML297 (*n* = 6) originated I/O curves for the first GABA_A_-dependent component of the fIPSP without significant differences with control animals for the 1st or 2nd stimulus. The linear fit of the scatter plot resulting after comparison of the 1st stimulus for ML297- vs. vehicle-injected animals showed a slope value > 1 (b = 1.20, *p* < 0.001; Figure 2E, center panel), suggesting a decrease in the inhibitory tone mediated by the GABA_A_ component.

Similarly, results for the group treated with A*β* + ML297 (Figure 2D, *n* = 13) showed no significant differences for the 1st or 2nd stimulus in the analyzed range of intensities (0.02–0.4 mA) when compared with the control (vehicle) group. The scatter plot showed a slope b = 0.90 (Figure 2D, center panel, *p* < 0.001), indicating an evolution of the inhibitory ionotropic postsynaptic component in the CA3–CA1 synapse parallel to that of the glutamatergic component (see [29]). Finally, mice injected with the selective GirK blocker tertiapin-Q (T-Q; *n* = 8) showed a decrease for the 2nd GABA_A_-dependent fIPSP amplitude, and the slope of the linear fit in the scatter plot (Figure 2F, center panel) had a value > 1 (b = 1.24, *p* < 0.001). These results show that the GABA_A_ receptor-dependent inhibitory activity was decreased in these animals, a change opposite to that observed for the glutamatergic activity (Figure S1).

Taken together, our results indicate that the amplitude of the fIPSP mediated by GABA_A_ receptors in CA3–CA1 dorsal hippocampus reflects the changes observed in the glutamatergic fEPSP in mice injected with vehicle, ML297, or the combination A*β* + ML297. However, in animals where GirK channels had been blocked by T-Q did lose the ability to adapt the inhibitory tone in relation to changes in the excitatory tone, as happens in A*β* injected mice.

### 2.2. GirK-Dependent Signaling Enhancement Prevents the Decrease in Synaptic Inhibition Caused by Aβ

**Figure 3:**
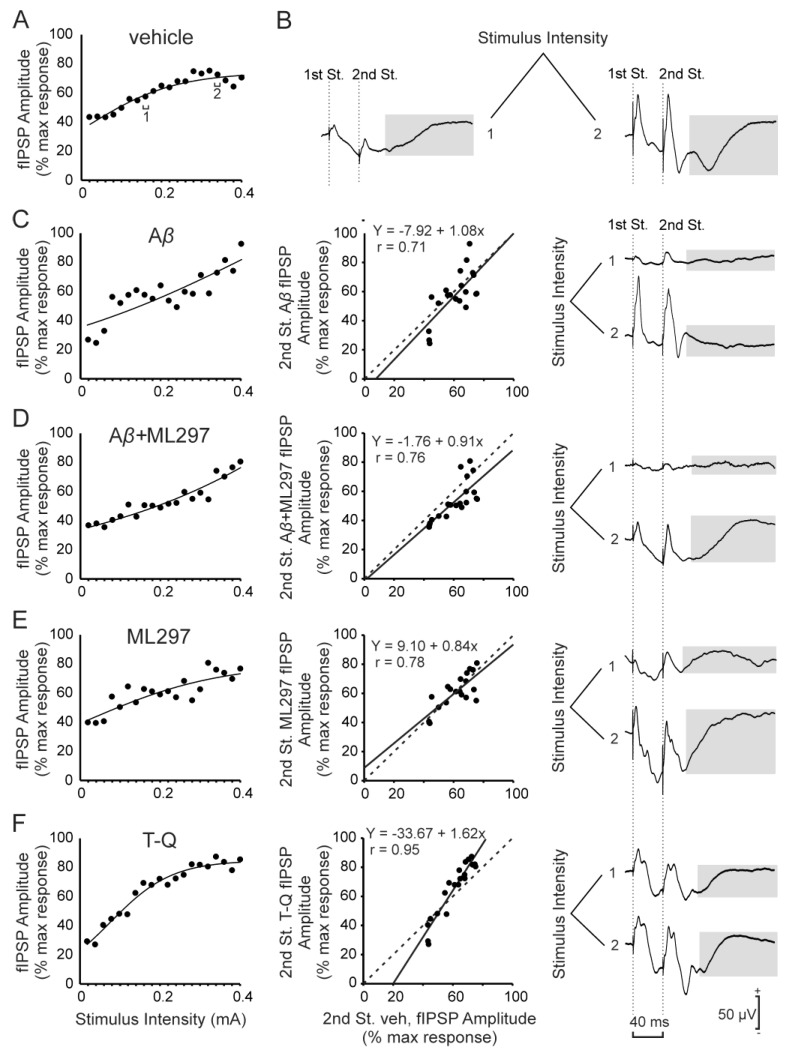
I/O curves for the GirK-channels-dependent inhibitory potential. (**A**) and left panel of (**C**–**F**), relationship between the intensity (mA) of pairs of stimuli (40 ms of interval between stimuli) applied to the Schaffer collaterals and the amplitude of the metabotropic GABAergic fIPSPs evoked in the CA1 region, corresponding to the second pulse. For each intensity of stimulation, circles represent the average of the response for all the animals of each experimental group. To facilitate the interpretation of the data, error bars have been omitted and the best sigmoid fit to the data for each group of animals has been illustrated (r ≥ 0.95 except for group A*β*, r = 0.845, and for group ML297, r = 0.857, *p* < 0.001). Center panel of (**C**–**F**), scatter plots and linear fits (continuous black lines) illustrating the amplitude values of fIPSP evoked by the second pulse in each experimental group vs. vehicle (control) group (x-axis, vehicle; y-axis, experimental group). Dashed lines represent the linear fit for the control conditions (vehicle vs. vehicle) and are equal in (**C**–**F**). The values of the slopes for these linear fits were compared with the control to study changes in the activity or tone of this inhibitory postsynaptic potential. Note changes induced by T-Q injection in the slope of the linear fit, which are opposite to the effect of ML297. (**B**) and right panel of (**C**–**F**) Averaged recordings (*n* = 5) representative of the fIPSPs evoked in the CA1 area after stimulation of the ipsilateral Schaffer collaterals with pairs of pulses (interval between stimuli of 40 ms) at two different intensities (1, 0.16 mA; 2, 0.34 mA; the intensities 1 and 2 are indicated in (**A**)). The highlighted area marks the metabotropic and GirK-dependent fIPSP subject of the analysis illustrated in this figure. St., stimulus.

Next, to further investigate the excitability control exerted by the inhibitory components of the CA3–CA1 synapse in alert mice, we studied the metabotropic fIPSP component, mediated by GirK channels (Figure 3B). This fIPSP was analyzed only in the response evoked by the 2nd stimulus, because its late latency (26–36 ms) and long duration made it impossible to measure in the time interval between the 1st and 2nd pulse (40 ms). In control mice (vehicle, *n* = 14), this fIPSP increased with current intensity (F_(19, 247)_ = 4.034, *p* < 0.001; Figure 3A). There were no significant differences between control animals and the other experimental groups in the evolution of this fIPSP throughout the analyzed intensities (F_(4,40)_ = 2.58, *p* > 0.05).

Comparing the linear fits for the scatter plots (Figure 3C–F, center panel), it can be observed that A*β*-injected mice (*n* = 6) showed only a subtle effect on this fIPSP, with a slope slightly > 1 (b = 1.08, *p* = 0.0004) (Figure 3C). This effect presented the same tendency as for the T-Q injections, although lower in magnitude. In fact, the slope for the mice injected with T-Q (*n* = 8) had a value > 1 (b = 1.62, *p* < 0.001) (Figure 3F), indicating a decrease of this inhibitory tone because of the blockage of GirK channels, and supporting the excess of neuronal excitability in the CA3–CA1 synapse previously found for these animals (Figure S1). The opposite effect is observed in the group injected with ML297 (*n* = 6), where the opening of GirK channels gave rise to a slope < 1 (b = 0.84, *p* < 0.001) (Figure 3E). Finally, the group injected with the combination A*β* + ML297 (*n* = 11) presented a slope < 1 (b = 0.91, *p* = 0.0001; Figure 3D, center panel), which suggests that the opening of GirK channels prevents the decrease in GirK inhibitory activity induced by A*β*.

### 2.3. Inhibitory LTP Mirrors Excitatory LTP in the CA3–CA1 Dorsal Hippocampal Synapse

**Figure 4:**
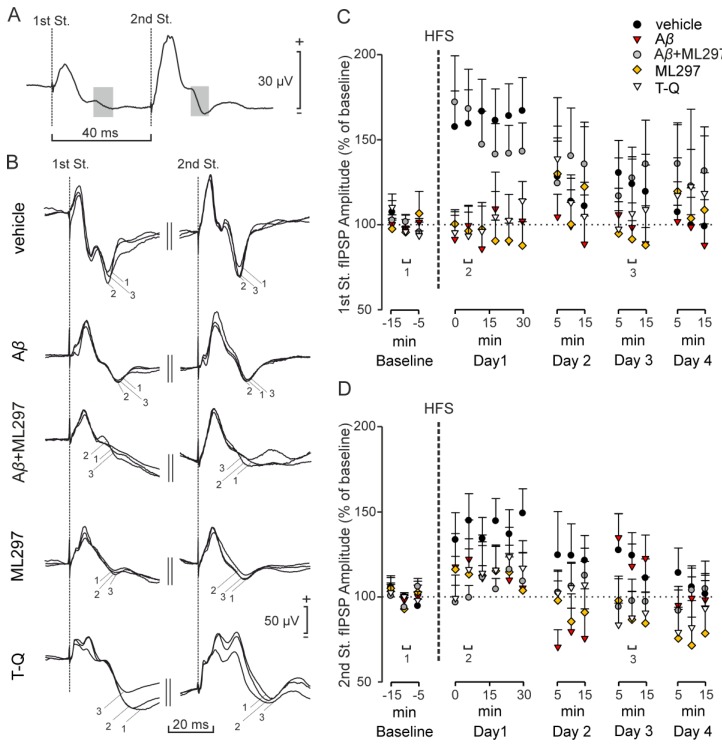
Evolution of the GABA_A_ receptor-dependent fIPSPs evoked in the CA1 area by stimulation of the Schaffer collaterals after an HFS session. The same intensity of stimulation was used before (baseline) and after the HFS protocol, and the amplitude of the ionotropic fIPSPs was measured. (**A**) Representative example of the analyzed fIPSP (the analyzed component is highlighted in gray). (**B**) Averaged examples (*n* = 15) of the metabotropic fIPSPs evoked in the CA3–CA1 synapse at different times: after injections but before HFS (1, baseline), 10 minutes after HFS (2) and 48 hours after HFS (3). Graphs show an illustration of the data (mean ± SEM) corresponding to the LTP of this inhibitory component of the postsynaptic response in control, A*β*-, A*β* + ML297-, ML297-, and T-Q-injected animals evoked by (**C**) the first and (**D**) the second pulse applied to the Schaffer collaterals. HFS, high-frequency stimulation; St. stimulus.

To ascertain whether LTP of inhibitory components is also present in alert animals, we induced LTP by high-frequency stimulation (HFS) of the hippocampal Schaffer collateral pathway in the different groups analyzed in the present work, and compared the evolution of fIPSPs evoked at the CA3–CA1 synapse in freely moving mice (Figure 4). In the fIPSP evoked by the 1st stimulus (Figure 4C), HFS induced LTP in the vehicle-injected mice (*n* = 12), but not in the A*β* (*n* = 6), ML297 (*n* = 5) and T-Q (*n* = 11) groups (F_(4,40)_ = 4.16, *p* = 0.007; *post hoc* Dunnett’s t vs. vehicle: A*β*, *p* = 0.040, ML297, *p* = 0.046, T-Q, *p* = 0.017). In a comparison of the values of fIPSP amplitude for the 1st stimulus with the baseline, control mice showed an average potentiation of 163 ± 7% (Figure 4C, black circles) during the 30 min following the HFS protocol (F_(2.12, 23.29)_ = 9.44, Greenhouse Geiser correction, *p* = 0.001). 

Interestingly, mice injected with A*β* + ML297 (*n* = 11) presented similar LTP values (Figure 4C, gray circles; base line vs. 30 min following HFS: F_(1.79,17.89)_ = 5.24, Greenhouse Geiser correction, *p* = 0.019) to those reached by the control (vehicle) group after the HFS session, with an average potentiation of 152 ± 8 % post hoc (Dunnett’s t vs. vehicle, *p* = 0.970). Therefore, our results show that an increased activity of GirK channels is able to restore GABA_A_-mediated inhibitory synaptic plasticity when A*β* blocks it.

In the fIPSP evoked by the 2nd pulse (Figure 4D), control (vehicle) mice (*n* = 12) also showed a significant potentiation, with an approximate value of 140 ± 6 % (Figure 4D, black circles), in the 30 min after the HFS protocol (F_(2.37, 26.03)_ = 4.92, Greenhouse Geiser correction, *p* = 0.012). However, LTP was not observed in A*β* (*n* = 5; F_(8,32)_ = 0.46, *p* = 0,873), ML297 (*n* = 5; F_(8,32)_ = 0.58; *p* = 0,786), or T-Q (*n* = 10; F_(1.52,13.70)_ = 1.10, Greenhouse Geiser correction, *p* = 0.34) groups, when compared with their respective baselines. No significant differences were found between the different experimental groups in their level of potentiation (F_(4, 39)_ = 1.34, *p* = 0.271). Mice injected with A*β* + ML297 (*n* = 12) did not present significant LTP (Figure 4D, gray circles; F_(3.29,36.24)_ = 1.57, Greenhouse Geiser correction, *p* = 0.211), with an average potentiation of 106 ± 3 %. The same was found when analyzing the fEPSP evoked by the same second stimulus (Figure S2). These results suggest that the GABA_A_ receptor-dependent fIPSP undergoes an LTP in the CA3–CA1 synapse that is proportional to the LTP of the glutamatergic fEPSP.

### 2.4. GirK-Dependent Signaling Undergoes LTP with a Late Appearance Latency after HFS

**Figure 5:**
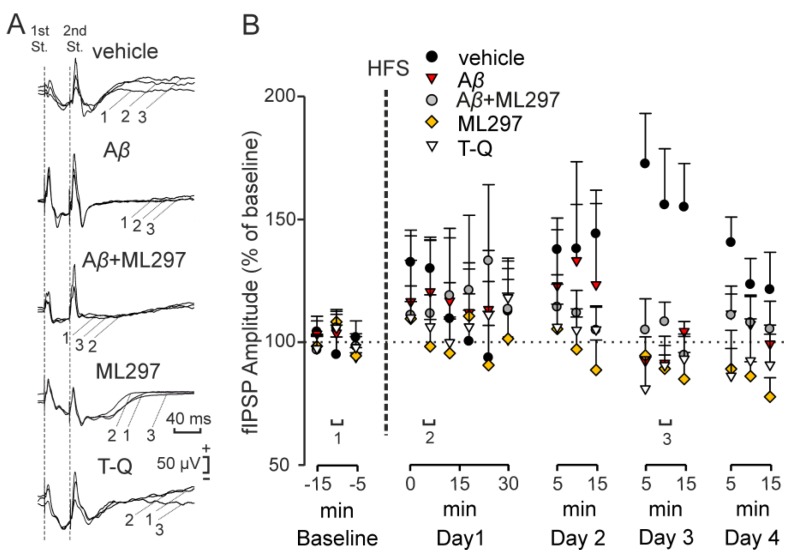
LTP of the GirK-channels-dependent fIPSP evoked by the second stimulus. Paired, squared, and biphasic pulses of 100 μs were used for the establishment of the baseline in freely moving mice, after which they were subjected to an HFS protocol. Next, the same stimulation used during baseline acquisition was applied to explore the evolution of the fIPSPs mediated by GirK channels and evoked in CA1 by the second stimulus applied to the Schaffer collaterals. (**A**) Representative examples (averaged, *n* = 15) of the metabotropic fIPSPs evoked in the CA3–CA1 synapse by 2nd pulse stimulation at different times: after injections but before HFS (1, baseline), 10 minutes after HFS (2) and 48 hours after HFS (3). (**B**) Illustration of the data (mean ± SEM) corresponding to LTP in control animals, and mice injected with A*β*, A*β* + ML297, ML297, and T-Q. HFS, high-frequency stimulation; St. stimulus.

As the existence of an LTP of GirK-dependent IPSPs in hippocampal slices has previously been reported [30], we wondered whether it would be found in alert animals. After HFS application to Schaffer collaterals in the different groups analyzed, we compared the evolution of fIPSPs evoked at the CA3–CA1 synapse in freely moving mice (Figure 5). Results after different drug injections differed from those observed for the glutamatergic fEPSP (Figure S2) and the ionotropic fIPSP (Figure 4). Mice injected with vehicle (*n* = 7) presented LTP, but it only became significant for the first time on Day 2 (F_(5, 30)_ = 4.32, *p* = 0.004), 24 h after HFS (Figure 5B, black circles). Maximum potentiation was reached 48 h after HFS, with values of 160 ± 11 % (F_(5, 30)_ = 6.34, *p* < 0.001), and remained significant on Day 4 (F_(5, 30)_ = 3.63, *p* = 0.011)—that is, 72 h after HFS. In contrast, there was an absence of GirK-dependent component LTP in mice injected with A*β* (*n* = 6), A*β* + ML297 (*n* = 12), ML297 (*n* = 5), and T-Q (*n* = 10) (*p* > 0.05 for all groups). All these results show that our HFS protocol is able to induce potentiation of GirK-dependent fIPSP. This potentiation is prevented by pharmacological manipulation of GirK channels and by A*β* in our in vivo model of early stages of AD. In addition, increased activity of GirK channels is not able to restore this form of synaptic plasticity when A*β* blocks it.

## 3. Discussion

In the dorsal hippocampus, neurotransmission balance between excitation and inhibition has capital importance for LTP, and subsequently, learning and memory processes [34]. In AD, alterations of the glutamatergic system has been widely studied, but inhibitory neurotransmission has raised a special interest only in recent years [8,35]. In this line, GirK channels have been shown to control resting membrane potential, because of their continuous basal activity [23,25], and to reduce the excess of synaptic excitation from distal dendrites to the soma of pyramidal cells [36]. Thus, we were prompted to analyze, in freely moving animals, the role of fIPSPs evoked in the CA1 region of the dorsal hippocampus after stimulation of the Schaffer collaterals, in a mouse model of early AD.

### 3.1. Role of GirK Channels in the Excitability of the Dorsal Hippocampus of Alert Mice

To study the role of GirK channels in the CA3–CA1 synapse we analyzed the excitability of the pathway in behaving mice. I/O curves for the inhibitory GABA_A_ dependent fIPSP reflected the same changes as those for the glutamatergic fEPSP when GirK currents are active. We previously showed that the increase in the activity of GirK channels by the administration of ML297 induces hippocampal hypoexcitability, together with a decrease in the inhibitory GABAergic tone [29]. It has been reported that the activation of presynaptic GABA_B_ receptors (of which GirK is an effector) with baclofen induces a decrease in postsynaptic GABA_A_ currents, in agreement with our observations [37]. Mice injected with A*β* prior to GirK activation (A*β* + ML297 group) showed a similar activity of the ionotropic fIPSP to that in control mice, as we observed when studying the fEPSP of the postsynaptic response [29]. These results indicate that changes in the amplitude of GABA_A_ dependent fIPSP are parallel to those of the glutamatergic fEPSP, meaning that an increase in neuronal excitation is accompanied by an increase in GABAergic inhibition, and vice versa. It could be a compensatory mechanism to maintain normal excitability levels in the CA3–CA1 synapse [38]. In fact, GABAergic remodeling has been observed in response to glutamatergic activity, since the activation of N-methyl-D-aspartate (NMDA) receptors can cause a temporary increase in postsynaptic GABA_A_ receptors [39].

However, in T-Q-injected animals, the blocking of GirK channel activity produced neuronal hyperexcitability by an increase in the excitatory tone (see Figure S1), without an increase in the GABA_A_-mediated component, which is in fact decreased. A decrease in GABA release and postsynaptic GABA_A_ receptors (in both number and activity) has also been reported in GIRK2 KO mice slices [40]. These results might indicate that blocking GirK channels disables one of the adaptive mechanisms needed to compensate the imbalances of neuronal excitability by modulating the inhibitory neurotransmission [4].

It is interesting to note that this GABA_A_-dependent fIPSP showed ambiguous results in A*β*-injected mice. On the one hand, A*β* administration increases the activity of this fIPSP evoked in the hippocampal CA1 region, manifested by a large increase in the amplitude of the 2nd stimulus. Neuronal hyperexcitability generated by acute exposure to A*β* or a brief application of convulsive stimuli have been shown to trigger compensatory mechanisms such as the sprouting of new GABAergic synapses and an increase in synaptic inhibition [1,41] in order to maintain normal synaptic activity and neuronal functionality. Although these compensatory modulations could be beneficial for the control of hyperexcitability, they also interfere with processes necessary for learning and memory, such as LTP [1]. On the other hand, the evolution of fIPSP evoked by the first stimulus in A*β*-injected animals is not altered, which might indicate that the inhibitory ionotropic GABAergic tone has not changed in these animals. Taken together, these results show that in A*β*-injected animals, the compensatory mechanisms needed for the maintenance of neurotransmission balance are impaired. This alteration could be due to a decrease in GirK channel activity induced by A*β* [31], although A*β* presented a much more subtle effect than the GirK channel inhibitor T-Q.

A*β* induced only a slight decrease in GirK-dependent inhibitory tone. The fact that A*β* modifies this fIPSP might indicate GirK channels as a molecular target for A*β*, as suggested using hippocampal slices [31,35,42] or primary cultures [43]. However, our in vivo results also showed that the effect of A*β* on this metabotropic fIPSP is lesser than the alterations induced when GirK channels are blocked by T-Q, in agreement with previous observations in vitro [31]. Such a weak blocking of GirK channels would not completely explain the A*β*-induced impairments on the glutamatergic fEPSP [29], greater than those induced by T-Q. This result supports the existence of different molecular targets for A*β* in the modulation of hippocampal neuronal excitability [6]. In addition, activation of GirK channels by ML297 after A*β* injections was able to prevent and overcorrect the decrease in the tone of this fIPSP, up to a higher level than for control animals, suggesting that the activating effect of ML297 on GirK channels is stronger than the blocking effect of A*β*. Nevertheless, although GirK channels are likely not the main target of A*β*, GirK activity enhancement in dorsal hippocampus would be an interesting therapeutic approach to restore the excitation/inhibition balance disrupted by A*β* [27,29].

### 3.2. GirK-Dependent Signaling Controls Synaptic Plasticity of Inhibitory Postsynaptic Responses in the Dorsal Hippocampus of Behaving Mice

In early stages of AD, synaptic plasticity deficits are present [44,45]. LTP is disrupted by the effects of A*β* both in vitro [46] and in vivo [29,47]. Evidence suggests that an increase in GirK-dependent signaling is detrimental for normal synaptic cognition and plasticity due to excessive inhibition [13,48,49], and the increased conductance of GirK in pyramidal neurons of the dorsal CA1 region is responsible for the higher stimulation threshold for LTP induction [25]. Studies on synaptic plasticity have predominantly focused on the mechanisms involved in the enhancement or depression of excitatory synapses. However, the correct functionality of the central nervous system requires a balance between inhibition and excitation, suggesting the existence of a similar modulation of inhibitory synaptic plasticity in the hippocampus [50,51]. Regarding GABAergic signaling, different observations about its plasticity process have been reported. It seems that theta burst stimulation (TBS) protocols, commonly used to trigger glutamatergic LTP, are capable of inducing a potentiation of the fIPSP dependent on GABA_A_ receptors in vitro. This potentiation lasts for approximately 30 min, and seems to be induced by mechanisms independent of NMDA-receptor signaling [50,52]. In contrast, HFS protocols, such as the one used in this study, seem to induce depression of the CA1 GABAergic synapses in vitro. This depression affects GABA_A_ and GABA_B_ receptor-dependent fIPSPs, so it could involve a decrease in the presynaptic release of GABA. The increase in intracellular Ca^2+^ after NMDA-receptor activation might trigger the synthesis and release of endocannabinoids by postsynaptic neurons. These endocannabinoids would cause a decrease in GABA release through their interaction with presynaptic cannabinoid receptor type 1 (CB1 receptors) located in hippocampal interneurons, and induce GABAergic long-term depression (LTD) [50,52].

Our work analyzes, for the first time, the synaptic plasticity of fIPSPs in vivo. We found, in contrast to the previous in vitro studies, a potentiation of the GABA_A_ dependent fIPSP after the presentation of an HFS protocol, mirroring glutamatergic LTP. We also observed that both modulation of GirK-dependent signaling and A*β* injections prevents this inhibitory LTP. In addition, the activation of GirK channels restored the LTP for the fIPSP caused by the first stimulus (but not the second) in A*β*-injected mice. These results show the presence of an LTP of the ionotropic GABAergic fIPSP in the CA3–CA1 synapse of the dorsal hippocampus, and that this synaptic plasticity depends on glutamatergic synaptic potentiation. Excitatory and inhibitory LTP might share, at least partially, the same molecular mechanisms. It has been reported that the activation of NMDA receptors, an event involved in the induction of glutamatergic LTP, can activate the traffic of GABA_A_ receptors to the dendrites of hippocampal neurons, causing an increase in the amplitude of this ionotropic fIPSP [53]. The main molecular mechanism by which these changes take place involves the participation of gephyrin, a scaffolding protein present in the GABAergic synapses, which is activated as a consequence of NMDA signaling, mediating the formation of new inhibitory clusters [54,55]. This would explain our observation of a GABA_A_ dependent fIPSP LTP equivalent to the excitatory LTP.

To date, very few studies about the LTP of the GirK-dependent fIPSP have been published. However, it has been reported that the same mechanism involving NMDA receptors and CaMKII for glutamatergic LTP causes an enhancement of GirK channel inhibitory currents in pyramidal CA1 neurons. Thus, the same signaling pathways necessary for excitatory synaptic plasticity might also induce long-term changes in synaptic inhibition [30,56]. In addition, activation of NMDA receptors has been shown to induce an increase in the surface expression of the GIRK1 and GIRK2 subunits [57]. In vitro studies have reported that NMDA-receptor activation triggers an increase in the density of GirK channels in cell membranes, their basal current, and their activation by A1 adenosine receptors (but not GABA_B_ receptors) [23,25,58]. In any case, the LTP of slow inhibition due to GIRK channels seems to represent one highly effective way of using excitatory synaptic inputs [30].

Our results revealed the existence of an LTP of the fIPSP dependent on GirK channels, in the response to the second stimulus applied in CA3, with a late appearance that peaked 48 h after the presentation of the HFS protocol. The temporal relationship between this potentiation and the glutamatergic LTP (increase in fIPSP potentiation concurred with the decrease in fEPSP amplitude [29]), supports other findings that present this plastic modulation of GirK activity as a necessary phenomenon for depotentiation of LTP in excitatory synapses [56,58]. Although depotentiation is usually defined as an impediment to inducing LTP, plasticity of GirK channel signaling might be involved in the extinction of the fEPSP potentiation to basal amplitude levels. Another consequence of GirK-dependent fIPSP potentiation would be the narrowing of the temporal window for the detection of excitatory stimuli [56], since late excitatory currents could be less effective due to the hyperpolarization caused by the increased GirK activity. Thus, synchronous stimuli would be detected more efficiently, with greater firing probability [30]. Therefore, plasticity in the activity of GirK channels seems to play an important role in the control of hippocampal performance.

We could detect the potentiation of the GirK-dependent fIPSP only in control animals. Pharmacological modulation of GirK channels, by altering the appropriate levels of channel activity, prevented this inhibitory LTP. A*β* ICV administration also blocked this form of synaptic plasticity, maybe as a consequence of the decrease in the activity of GirK channels [31], and also because of the molecular mechanisms shared with glutamatergic LTP, which is affected by the action of A*β* in vivo [11,29,47,59]. In fact, the activation of GirK channels was not able to restore the LTP of this fIPSP in the mice injected with A*β*, as observed for the LTP of the glutamatergic fEPSP and ionotropic fIPSP evoked by the second stimulus (that is, the stimulus in which the analysis of the GirK-dependent fIPSP was performed). These results show the close relationship and coordination between excitatory and inhibitory activity and plasticity, in the hippocampal synapses.

In summary, the temporal evolution of the three studied fPSPs (early appearance of LTP for glutamate and GABA_A_-dependent fPSPs, and late appearance for the GirK-dependent fIPSP) shows the necessity that excitatory and inhibitory LTPs are orchestrated in a coordinated manner in the CA3–CA1 synapse of dorsal hippocampus in alert, freely moving mice, because this temporal organization seems to be essential for learning and memory processes [29,32,33].

## 4. Materials and Methods

### 4.1. Subjects

C57BL/6 male adult mice (3–5 months old; 28–35 g; *n* = 90) obtained from an official supplier (Janvier Labs, Saint Berthevin, France) were used for these experiments. Before surgery, animals were housed in separate cages (*n* = 5 per cage), on a 12 h light/dark cycle with constant ambient temperature (21 ± 1 °C) and humidity (50 ± 7 %). Food and water were available ad libitum. All experiments were performed in accordance with European Union guidelines (2010/63/EU) and with Spanish regulations for the use of laboratory animals in chronic experiments (RD 53/2013 on the care of experimental animals: BOE 08/02/2013), and approved by the local Ethics Committees of the Universities of Castilla-La Mancha and Pablo de Olavide.

### 4.2. Surgery for Chronic Recordings and ICV Injections in Alert Mice

Surgery for chronic recordings and ICV injections followed in the present study have been previously described [29]. Briefly, subjects were anesthetized with 4 % chloral hydrate and implanted with bipolar stimulating electrodes made from 50 μm, Teflon-coated tungsten wire (Advent Research Materials, Oxford, UK), aimed at the right Schaffer collateral-commissural pathway of the dorsal hippocampus (2 mm lateral and 1.5 mm posterior to bregma; depth from brain surface, 1.0–1.5 mm [60]), and with recording electrodes aimed at the ipsilateral stratum radiatum underneath the CA1 area (1.2 mm lateral and 2.2 mm posterior to bregma; depth from brain surface, 1.0–1.5 mm [60]) (Figure 1A). The final position of the hippocampal electrodes was determined by evaluating the field potential depth profile evoked responses presented at the Schaffer collateral pathway [61]. A bare silver wire (0.1 mm) was affixed to the skull as a ground. All electrodes and the ground were connected to a 6-pin socket that was fixed to the skull with dental cement [61].

Animals were also implanted chronically with a blunted, stainless steel, 26-G guide cannula (Plastic One, Roanoke, VA, USA) in the contralateral ventricle (0.5 mm posterior to bregma, 1.0 mm lateral to midline, and 1.8 mm below the brain surface [60]), in order to perform the ICV administration of drugs included in this study (Figure 1B) and preserve the contralateral CA3–CA1 pathway intact to perform electrophysiological recordings in alert mice. Mice were allowed a week for recovery before the experimental sessions. ICV injections of 3 μL volume were performed in freely moving mice. Injections were carried out at a rate of 0.5 μL/min with the help of a 33-G internal cannula and a motorized Hamilton syringe. Before removal, the internal cannula was left five extra minutes inside the guide cannula to avoid any backflow. Electrophysiological recordings were successfully obtained 24 h after ICV drug injections.

### 4.3. Input/output Curves

fPSPs were recorded from alert behaving mice with Grass P511 differential amplifiers through a high-impedance probe (2 × 1012 Ω, 10 pF). For fPSPs, electrical stimuli presented to Schaffer collaterals consisted of 100 μs, square, biphasic pulses presented alone, paired, or in trains. For the construction of I/O curves, 24 h after ICV drug injections, freely moving mice were placed inside a small box (5 cm× 5 cm× 5 cm) and connected to the acquisition system. Paired, square, biphasic pulses of 100 μs duration, with a fixed interstimulus interval of 40 ms, were applied through the stimulating electrode to Schaffer collaterals, at increasing intensities ranging from 0.02 mA to 0.4 mA, with increments of 0.02 mA (i.e., 0.02 mA, 0.04 mA, 0.06 mA, …, 0.4 mA). Changes in stimulation intensity were manually modulated (ISU 200 BIP, Cibertec, Madrid, Spain). For each intensity, the stimulation was repeated a minimum of five times. fPSPs evoked in CA1 were recorded. Two different inhibitory postsynaptic potentials (fIPSP, see “fIPSP analysis”) were averaged (*n* ≥ 5), and their amplitude was analyzed for all stimulus intensities. The evolution of the curves was visualized during the experiment in an oscilloscope (Tektronix TDS 3014C, Salem, OR, USA).

### 4.4. Long-Term Potentiation

LTP induction took place 24 h after the ICV injections, with alert, freely moving animals placed inside a small box (see input/output curves). Paired, square, biphasic pulses of 100 μs duration (interstimulus interval of 40 ms) were applied at a constant intensity necessary to evoke ~35% of the maximum fEPSP response in the CA1 hippocampal region. An additional criterion for the selection of pulse intensity for the induction of LTP was that the second stimulus should provoke a field postsynaptic potential of greater magnitude (≥150 %) than the first pulse. A baseline was established by the stimulation of Schaffer collaterals at 0.05 Hz for 15 min, prior to LTP induction. After that, mice were presented with an HFS session [61], consisting of five trains of 100 ms duration at 100 Hz, applied at a rate of 1/s. These trains were repeated six times at intervals of 1 min ─ that is, a total of 300 pulses presented. The duration and intensity of the pulses were not modified with respect to the baseline. Post-HFS recordings were acquired for 30 min after the HFS session, and for 15 min on the three following days, using the same stimulation as for the baseline, in order to study the evolution of LTP. For the analysis, fIPSPs were averaged (*n* = 15), and their amplitudes were measured. All the data obtained from the CA1 hippocampal region were normalized using the fIPSP values collected during the baseline as 100% of the response.

### 4.5. fIPSP Analysis

The inhibitory postsynaptic potentials (fIPSPs) analyzed were identified according to their latency of appearance (see Figure 1C). Stimulation of the Schaffer collaterals evoked a glutamatergic wave with a latency of 2.25–4 ms as the main excitatory component of the postsynaptic response, closely followed by an inhibitory, GABA_A_ receptor-dependent postsynaptic potential with a latency of 12–15 ms. A slow fIPSP appearing 26–36 ms after stimulation was identified as the GPCRs and GirK-channel-dependent inhibitory component of the response [32,33]. This fIPSP was analyzed only after the second pulse, due to its late appearance.

### 4.6. Drugs

All drugs selected for this study were purchased from Abcam (Bristol, UK) and dissolved in PBS (vehicle) with the aid of a vortex and/or sonicator. As described elsewhere [29], drugs were dissolved in 3 μL of vehicle. Animals were randomly assigned to the different experimental groups before performing the injections. 3 μg of A*β*_1–42_ were dissolved (0.22 mM) and injected through the guide cannula at a rate of 0.5 μL/min. To create a model of local acute amyloid pathology in the dorsal hippocampus, mice were injected ICV. with A*β*_1–42_, thus generating a non-transgenic model in vivo. A*β*_1–42_ was dissolved in vehicle and incubated for 1 h at room temperature in order to form the highly toxic prefibrillar oligomers [62,63] present in early stages of AD. ML297, a specific activator of GIRK1-containing GirK channels, was dissolved in vehicle at a concentration of 1.5 mM, and administered at the same rate as A*β* [64]. These quantities were determined based on preliminary tests and the existing bibliography [63]. For the subjects where a combination of A*β*_1–42_ and ML297 was administered, sequential 3µL-injections of each drug were made at 15 min intervals (A*β* administration took place first, and ML297 was injected 15 min later). T-Q was used for the selective blocking of GirK channels, after being dissolved in vehicle at a concentration of 244.6 μM. This concentration was established based on preliminary tests.

### 4.7. Statistics

Data are represented as the mean ± standard error. All statistical calculations were performed using the SPSS Statistics software (SPSS Inc., Chicago, IL, USA). When the distribution of the variables was normal, the acquired data were analyzed with the repeated measures ANOVA test, where the treatment was the inter-subject variable, and the intensity of stimulation (curves I/O) or time (LTP) was the intra-subject variable. The Greenhouse Geiser correction was used on the occasions in which the sphericity of the sample was not assumed. Post hoc analyses (Dunnett’s t) were performed to further study the significant differences between experimental groups. If the Levene test for the homogeneity of variances was significant, the data were normalized by logarithmic transformation (Ln). For scatter plots, we calculated Pearson’s correlation factor and the level of signification. Statistical significance was established at *p* < 0.05.

## Supplementary Materials

Supplementary materials can be found at https://www.mdpi.com/1422-0067/20/5/1168/s1.

## Abbreviations

A*β*	amyloid-*β*
AD	Alzheimer’s disease
CB1	cannabinoid receptor type 1
fEPSP	field excitatory postsynaptic potential
fIPSP	field inhibitory postsynaptic potential
fPSP	field postsynaptic potential
GirK	G-protein-gated potassium channels
GPCR	G-protein-coupled receptors
HFS	high-frequency stimulation
ICV	intracerebroventricular
LTD	long-term depression
LTP	long-term potentiation
NMDA	N-methyl-D-aspartate
TBS	theta burst stimulation
T-Q	tertiapin-Q
