# Supplementary material for: Role of GirK Channels in Long-Term Potentiation of Synaptic Inhibition in an In Vivo Mouse Model of Early Amyloid-β Pathology
Source: Int J Mol Sci. 2019 Mar 7;20(5):1168. doi: 10.3390/ijms20051168 (PMC6429279; doi:10.3390/ijms20051168)
Supplement: Supplementary file 1 [file ijms-20-01168-s001.pdf]

Supplementary Materials:

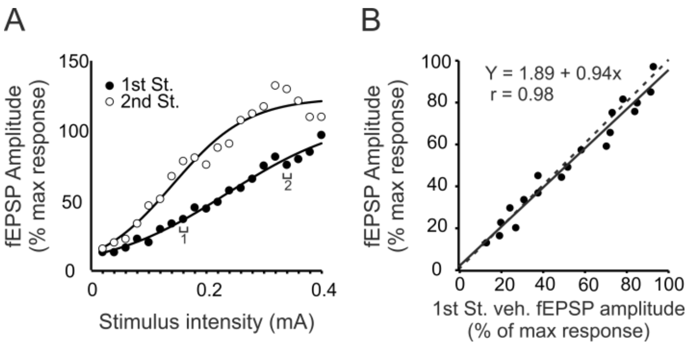

Figure 1. I/O curves for the glutamatergic fEPSP in T-Q-injected mice. (**A**) Relationship between the intensity (mA) of pairs of stimuli (40 ms of interval between stimuli) applied to the Schaffer collaterals and the amplitude of the glutamatergic fEPSPs evoked in the CA1 region, corresponding to the first (black dots) and second (white dots) pulse. For each intensity of stimulation, circles represent the average of the response for all the animals of each experimental group. To facilitate the interpretation of the data, error bars have been omitted and the best sigmoid fit to the data has been illustrated. (**B**) Scatter plots and linear fit (continuous black line) illustrate the amplitude values of fEPSP evoked by the first pulse in T-Q-injected animals vs. control (x-axis, vehicle; y-axis, T-Q group). Dashed lines represent the linear fit for the control conditions (vehicle vs. vehicle). Observe how the slope is < 1 (b = 0.94), in agreement with excitability in the CA3-CA1 synapse.

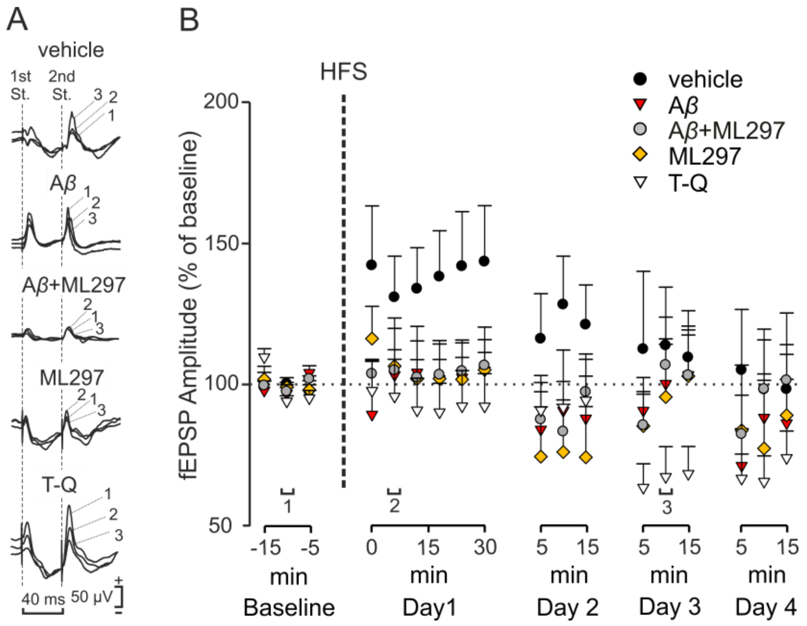

Figure S2. LTP of the fEPSP evoked by the second stimulus in the hippocampal CA3-CA1 synapse. Paired pulses (40 ms interstimulus interval) were used for the establishment of the baseline in freely moving animals. Next, an HFS protocol was presented. After that, the same stimulation used during baseline recordings was applied to study the changes in amplitude for the fEPSPs evoked in CA1 by the second pulse applied to the Schaffer collaterals. (**A**) Representative examples (averaged, *n* = 5) of the fEPSPs evoked at the CA3-CA1 synapse by stimulation with the 2nd pulse at different times: after injections but before HFS (1, baseline), 10 min after HFS (2), and 48 h after HFS (3). (**B**) Illustration of the data (mean ± SEM) corresponding to LTP induced in the control animals, and mice injected with A*β*, A*β* + ML297, ML297, and T-Q.
